# Genome-wide identification and interactome analysis of members of two-component system in Banana

**DOI:** 10.1186/s12864-019-6050-1

**Published:** 2019-08-27

**Authors:** Yogeshwar V. Dhar, Deepika Lakhwani, Ashutosh Pandey, Shikha Singh, Prabodh K. Trivedi, Mehar H. Asif

**Affiliations:** 1 CSIR-National Botanical Research Institute (CSIR-NBRI), Rana Pratap Marg, Lucknow, 226001 India; 2 Academy of Scientific and Innovative Research (AcSIR), Ghaziabad, 201002 India; 3 National Institute of Plant Genome Research, Aruna Asaf Ali Marg, P.O. Box No. 10531, New Delhi, 110 067 India

**Keywords:** Banana, Ethylene, Fruit ripening, Signal transduction, Two-component system

## Abstract

**Background:**

Ethylene signal transduction in plants is conducted by the two-component system (TCS) which consists of histidine kinase (HK), histidine phosphotransferase (HPT) and response regulators (RRs). This system plays an important role in signal transduction during various cellular processes, including fruit ripening and response to multiple environmental cues. Though members of TCS have been identified in a few plants, no detailed analysis has been carried out in banana.

**Results:**

Through genome-wide analysis, we identified a total of 80 (25 HK, 10 HPT and 45 RR) and 72 (25 HK, 5 HPT and 42 RR) TCS genes in *Musa acuminata* and *Musa balbisiana* respectively. The analysis of identified genes revealed that most of the genes are highly conserved however; there are subtle divergences among various members. Comparative expression analysis revealed an involvement of a set of TCS members during banana fruit ripening. Co-expression network analysis identified a working TCS module with direct interactions of HK-HPT and RR members. The molecular dynamics analysis of TCS module showed a significant change in structural trajectories of TCS proteins in the presence of ethylene. Analysis suggests possible interactions between the HK-HPTs and RRs as well as other members leading to banana fruit ripening.

**Conclusions:**

In this study, we identified and compared the members of TCS gene family in two banana species and showed their diversity, within groups on the basis of whole-genome duplication events. Our analysis showed that during banana fruit ripening TCS module plays a crucial role. We also demonstrated a possible interaction mechanism of TCS proteins in the presence and absence of ethylene by molecular dynamics simulations. These findings will help in understanding the functional mechanism of TCS proteins in plants in different conditions.

**Electronic supplementary material:**

The online version of this article (10.1186/s12864-019-6050-1) contains supplementary material, which is available to authorized users.

## Background

The two-component system (TCS) is one of the most evolutionarily conserved signalling cascade present from prokaryotes to eukaryotes. In bacteria, the TCS follows simple His-to-Asp auto-phosphorelay [1], having a sensory histidine kinase with an N-terminal input domain and a C-terminal kinase domain with an invariant histidine residue. In plants, TCS consists of three members for the signal transduction comprising histidine kinase or hybrid HK (containing both sensory as well as receiver domain), histidine containing phosphotransmitter (HPT) and response regulator (RR) proteins. The HKs were extensively studied in *Arabidopsis* and are classified into three subgroups; cytokinin receptors, ethylene receptors and phytochromes. In addition, three additional histidine kinases (CKL1, AHK5, AHK1) were identified in *Arabidopsis* which do not belong to these groups. The protein structure of histidine kinase contains a hybrid sensor HK containing variable input domain, N-terminal transmembrane domain and a transmitter domain with a conserved His residue site of autophophorylation with fused Rec domain [2]. Ethylene receptors and phytochromes lack key residues in conserved HK domain and are called diverged HKs of two-component elements [3–5]. Three cytokinin receptors (AHK2, AHK3, AHK4) contain a structure of cyclase/HK-associated sensory extracellular (CHASE) domain which is the putative site for the recognition of cytokinin [6]. AHK4 has dual activities depending on availability of cyokinin. In the presence of cytokinin, it acts as kinase and phosphorylates HPT domain, while in the absence, acts as phosphatase and thus dephosphorylates HPT. Ethylene receptors (ETR1, ETR2, ERS1, ERS2 and EIN4) contain an ethylene-binding domain whereas phytochromes (PHY A, B, C, D and E) share a chromophore-binding domain (PHY).

Histidine containing phosphotransmitters (HPTs) can mediate the transfer of a phosphate group from the Rec domain of AHK to the Rec domain of the response regulator [7]. It contains a highly conserved motif xHQxKGSSxS. However, there is an additional group in HPTs known as pseudo-His-containing phosphotransfer protein which lacks the conserved histidine residue. This group is unable to function as phosphotransmitter protein and participates in cytokinin signalling by inhibiting phosphorelay from phosphorylated AHP1 to ARR1.

The RRs have been classified into two subgroups, Type-A RRs and Type-B, according to their amino acid sequences and conserved domains. Type-A RRs are primary cytokinin response proteins consisting of a Rec domain along with a short C-terminal extensions. The Type-B RRs are composed of Rec domain and a large C-terminal extension with a GARP (Golden/ARR/Psr1) motif of 60 amino acids [8]**,** which are closely related with the MYB DNA-binding domain family. Like diverged HKs, there are some pseudo-RRs (PRRs) which are referred as the diverged RRs and have conserved Asp for phosphorylation [9]. Clock PRRs contains distinct and specific CCT motif in their C-terminal extensions and functions in the regulation of the circadian rhythms [10]. Due to the important role of the TCS gene families in various plant processes, members of this family have been identified in several plants. However, no effort has been made to identify members of this vital signalling module from banana.

Bananas are perennial monocot herbs of the order Zingiberales and considered as economically important fruit crop world-wide due to its nutritional values and good yield. The establishment of the whole-genome sequence of the banana species, *M. acuminata* and *M. balbisiana,* by Global *Musa* Genomic Consortium [11, 12], enabled us to study the genome-wide evolution and divergence of the different gene family members. In this study, the TCS gene families from *Musa* spp. were identified at the genome-wide level and functionally analysed. Phylogenetic studies were carried out to classify the identified genes on the basis of their structure and function. In addition, their expression and interaction within different members have been identified. The analysis revealed specific interactions of TCS members leading to banana fruit ripening.

## Results

### Identification of TCS proteins in *Musa* spp.

A total of 80 and 72 TCS genes were identified in *M. acuminata* and *M. balbisiana* respectively through a profile Hidden Markov Model (HMM) developed using 238 TCS genes from 9 plant species and HMMERv3 [13]. After removal of all the partial and redundant sequences 25 HK, 10 HPT and 45 RR genes in *M. acuminata*, and 25 HK, 5 HPT and 42 RR genes in *M. balbisiana* were identified and analysed in this study. All the identified members of the TCS gene families were named according to the proposed nomenclature scheme for the two-component system [14]. TCS gene family members have already been studied in a few plants including *Arabidopsis thaliana, Oryza sativa, Glycine max, Physcomitrella patens, Zea mays, Brassica rapa, Nelumbo nucifera.* Results suggest a variation in the total number of genes (Additional file 1: Table S1) in different plants with highest members in *G. max* [15] followed by *M. acuminata* and *M. balbisiana*. Our analysis suggests presence of the largest number of TCS genes in *Musa* spp. among all the reported monocots. Interestingly, the increase in the TCS members is mainly due to the expansion of the HK protein family [16–18].

### HK protein family in *Musa* spp.

*M. acuminata* and *M. balbisiana* have the largest number of HK family members (25) as compared to other monocot species. The HK family is further classified into three subgroups, CHK/HK, PHY and ETR. Out of the 25 HK genes identified in *M. acuminata,* 11, 6 and 8 members were classified as CHK/HK, PHY and ETR subgroups. Similarly, *M. balbisiana* genome contained 12 CHK/HK, 4 PHY and 9 ETR subgroup members. HK/CHK subgroup members either possess simple histidine kinase domain or a CHASE associated HK domain (CHK). In *M. acuminata,* 2 HK (*MaHK2, MaHK4*) and 9 CHK whereas in *M. balbisiana* 3 HK (*MbHK1, MbHK2 and MbHK4*) and 9 CHK proteins contain HK domain followed by HATPase_c domain. Of these, MbHK1 did not contain well conserved Rec domain. The CHK proteins have 2–3 trans-membrane (TM) domains surrounding CHASE domain followed by HK, HATPase_c and Rec domains. Similar structure of the HK/CHK was also observed in other plants. Apart from the presence of the standard domain architecture, variations in the structure of the CHKs in *Musa* spp. were observed which might be due to their specific functions.

**Fig. 1:**
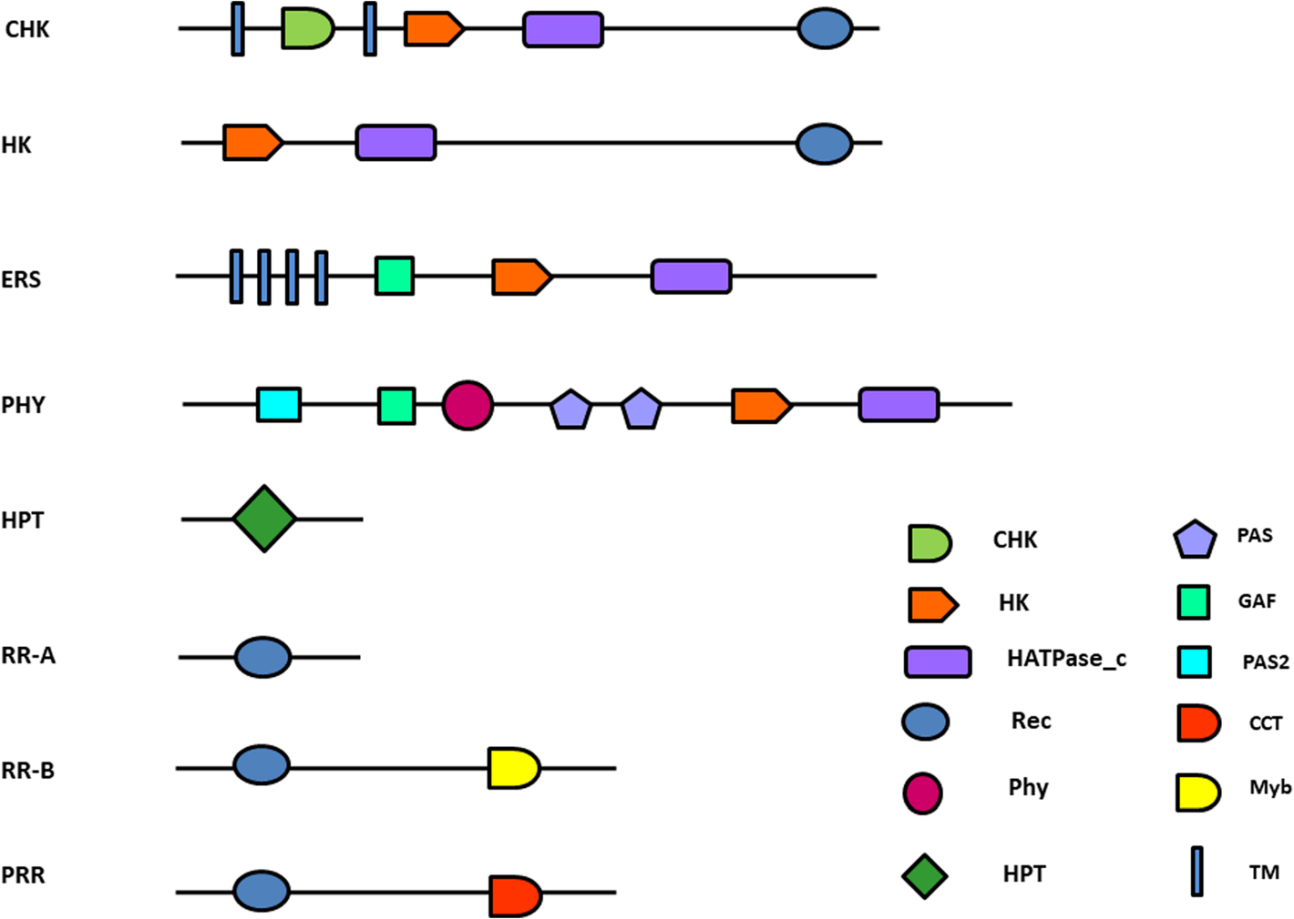
Standard domain architecture of the TCS proteins. The standard domain architecture of TCS proteins in *M. acuminata* and *M. balbisiana* was computed based on CDD and SMART protein domain analysis tool. Domain names are given different shapes and included in each figure as per their presence. Figures are non-scaled

All the CHKs of *M. acuminata* showed the standard domain architecture however, a few differences in the signal peptide of CHKs of *M. balabisiana* were observed. MbCHK2.D showed a variation in the position of the TM domain where presence of two TM domains has been observed, one on N-terminal and the other on C-terminal regions. MbCHK3.A and MbCHK3.B showed the presence of initial signal peptide, 3 TMs, 1 HK, 1 HATPase_c and 2 repeated Rec domains. Interestingly, MbCHK3.C had 1 TM, CHASE domain followed by HK and Rec domains, showing the absence of HATPase_c domain (Fig. 1, Additional file 2: Figure S1). Additional TM domains present in the CHK proteins have been shown to play an important role in the membrane associated signal transduction in plants [16, 19].

Ethylene receptors have a common domain structure containing 3–4 TM, GAF, HK, HATPase and Rec domains. In *Musa* spp., 8 and 9 ethylene receptors were identified in *M. acuminata* and *M. balbisiana* respectively. The ETR1 proteins in *Musa* spp. (MaETR1.B, MaETR1.C, MbETR1.B and MbETR1.C) had all the standard domains present in ethylene receptors. MaETR1.A lacked the GAF domain, whereas its counterpart in MbETR1.A retained the same. The ERS proteins in *M. acuminata* and *M. balbisiana* show quite a few differences in the presence of domains. The general domain organization of ERS includes 3–4 TM followed by GAF, HK and HATPase_c domains. This domain architecture was also found in MaERS1.B, MbERS1.A and MbERS1.B. The diverged domain organization was also observed in MbERS1.D and MbERS2, where TM and GAF were present in MbERS1.A but absent in MbERS2. The presence of a few additional domains was observed in MbERS1.C in addition to standard ERS domain structure. These additional domains include HPT, Act-Frag_Cataly and PTPc_DSPc with Zinc_fingers. The MaERS1.A also lacked the TM domain, which is essential for ethylene binding and subsequent signalling cascade; hence, MaERS1.A might not be playing an important role in the ethylene perception (Fig. 1, Additional file 2: Figure S1).

Phytochrome receptors are characterized by the presence of a chromophore binding group and 2 PAS domains. Six and four *PHY* genes were identified in *M. acuminata* and *M. balbisiana* respectively. All the identified PHY in *M. acuminata* and *M. balbisiana* showed the presence of standard phytochrome domain architecture, which includes HK, GAF, chromophore binding PHY domain and 2 PAS domains. The domains PHY, GAF and PAS play important roles in responding to red and far-red light signals during plant growth and development in *Arabidopsis* [4]. Only MaPHYC.B lacked the GAF domain (Fig. 1, Additional file 2: Figure S1).

### HPT protein family in *Musa* spp.

Histidine containing phosphotransmitters (HPT) are the signal mediators in multistep His-to-Asp phosphorelay and originated in plants [7]. These are highly conserved in *Musa* spp., having from 150 to 155 amino acid residues including HPT domain. These sequences showed 50–80% identity with rice and maize and 30–40% identity with Arabidopsis. In *M. acuminata,* 10 HPTs were identified of which 3 are pseudo-HPTs. In *M. balbisiana,* 7 HPTs were identified of which 1 was a pseudo-HPT. In *M. balbisiana,* 4 different domain architectures for HPT proteins were identified of which first architecture contains 3 small HPT stretches of 35–45 amino acid residues and one larger HPT stretch of 85 amino acid residues, with HK, MYB and TFIID/TAF domains. MbHP3 contained TM, 2 cytochrome P450 and HATPase_c with HPT domain. Another interesting domain organization was observed in MbHP7, where HPT domain was found with TM, P450 and STAT_int domains which have role in innate and host acquired immune responses. In MbHP8, HPT domain was observed with OmpH and Duff domain (Fig. 1, Additional file 2: Figure S1). Interestingly, the presence of these domains in HPT proteins was not observed or reported in earlier TCS component studies in other plants.

### RR protein family in *Musa* spp.

RRs works as a signal executor in the TCS signalling cascade and are divided into four groups based on their domain organization. Sequences containing Rec domain are classified as RR-Type-A whereas those with Rec and MYB (GARP, SANT motif) domains are classified as RR-Type-B. In addition, sequences which contains pseudo Rec domain (lacking conserved Asp residue) with CCT (Co, Col and Toc) domain are considered as PRR. A total of 46 *RR* genes were identified in *M. acuminata* of which 20 are Type-A, 14 Type-B, one Type-C and 11 PRR. In *M. balbisiana,* 42 *RR* genes are identified, 18 Type-A, 13 Type-B and 11 PRRs. These sequences showed 20–60% identity with Arabidopsis while 35–75% of identity was observed with rice. All the TCS elements of *Musa* spp. showed comparatively higher identity with rice and maize as compared to Arabidopsis.

All the sequences containing Rec domain only, were identified as Ma/Mb RR-Type-A ranging from 141 to 229 amino acid residues. One member of this group in *M. acuminata*, MaRR9, was identified with Rec domain followed by DUFF domain having sequence length of 688 amino acid residues. In comparison with *M. acuminata*, *M. balbisiana* RR-Type-A proteins showed presence of additional domains. MbRR1 sequence contains PPR1 domain in repetitive numbers. MbRR2, apart from Rec domain, contains RAS_bdg2 domain. Similarly, MbRR14 of 351 amino acid residues shows the presence of Rec domain with cytokinin receiver CHASE and apoptosis related CASc domains and MbRR16 showed presence of CPDc domain along with Rec domain. Members of Type-B RR in *M. acuminata* were observed with standard domain composition of Rec followed by MYB domain. However, in *M. balbisiana*, the Type-B sequences were observed with stretches of additional functional domains which are mostly related to the apoptosis or defence against stress/pathogen. ATG19, Ellicitin, CARD and Antimicrobial21 are a set of such domains. Presence of these domains indicates the responsiveness/sensitivity towards defence response during different stress conditions. Presence of such domains in the Type-B RRs also indicates their possible role in initiating/regulating ethylene mediated defence pathway in *Musa* plants (Fig. 1, Additional file 2:Figure S1).

### Phylogenetic analysis of TCS members

**Fig. 2:**
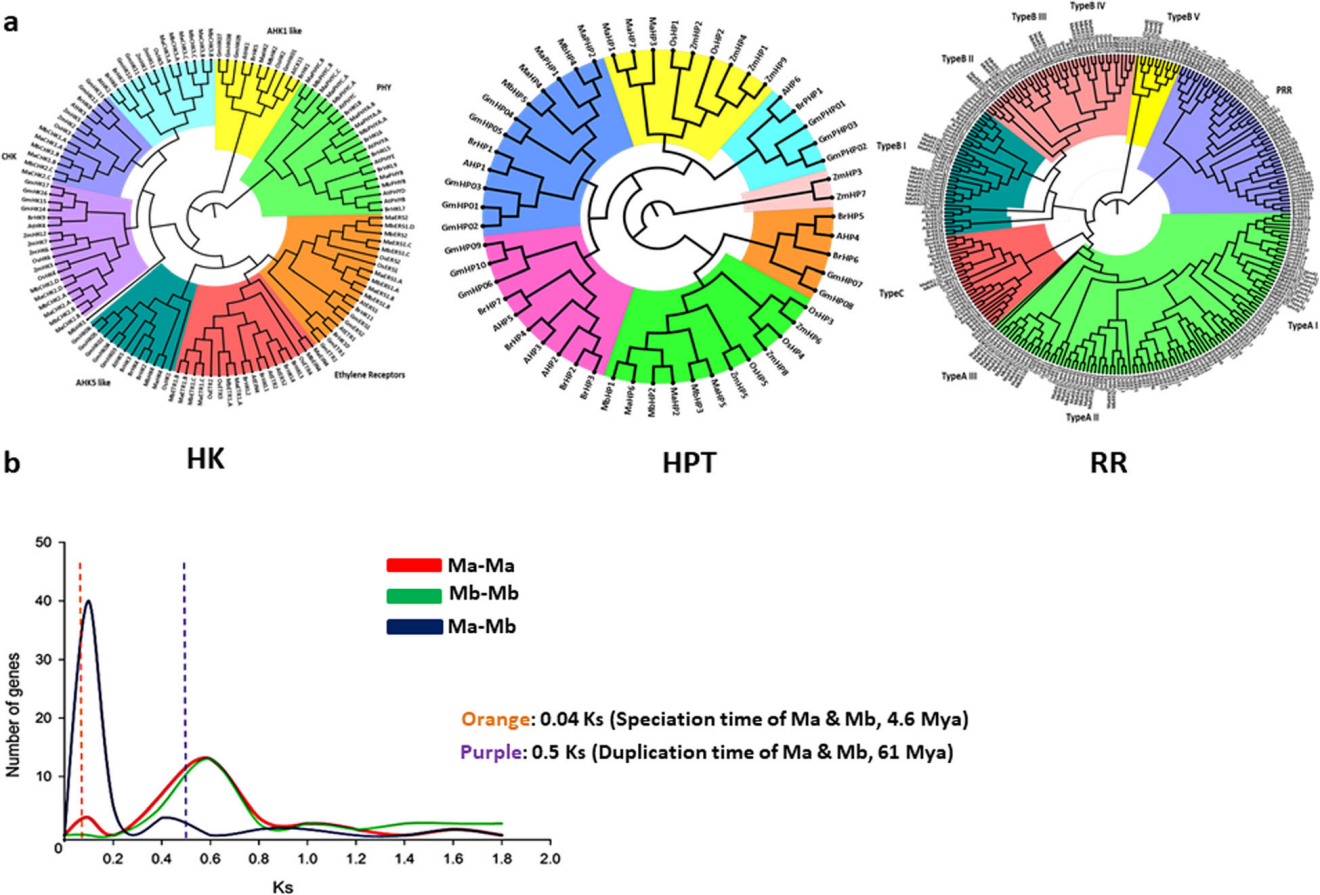
Phylogeny and substitution analysis of TCS genes in banana. **a** Unrooted phylogenetic trees for histidine kinase (HK), histidine containing phosphotransmitters (HPT) and response regulators (RR) of *M. acuminata* and *M. balbisiana* with *Arabidopsis*, *Oryza sativa*, *Zea mays*, *Brassica rapa* and *Glycine max* sequences, generated using maximum likelihood with 1000 bootstrap. Colours represent different sub clades of tree with group names. **b** Synonymous substitution frequency plot for the reciprocal hits of the TCS genes of *M. acuminata* and *M. balbisiana*. Red and green lines show duplication of genes for *M. acuminata* and *M. balbisiana* respectively. Blue line represents the divergence between the genes of *M. acuminata* and *M. balbisiana*. Vertical orange bar is representing the speciation time between two genomes, whereas the purple bar showing duplication between the *M. acuminata* and *M. balbisiana*

To study the phylogenetic relationship among TCS family members, protein sequences encoded by different genes from *M. acuminata*, *M. balbisiana*, *A. thaliana*, *O. sativa* and *G. max* were aligned using the ClustalX program and used to construct the phylogenetic tree using MEGA7 [20]. A total of 48 HK, 33 HPT and 156 RR sequences were used to construct the phylogeny. The HKs grouped into 8 subfamilies namely cytokinin receptor, ethylene receptor, PHY-like, CKI1-like, CKI2/AHK5-like, AHK1-like and PDK-like. In most of the groups of HK, monocots grouped together, however in a few cases there were no monocot- or dicot-specific groups. In nearly all the groups, *M. acuminata* and *M. balbisiana* proteins grouped together. Except for PHY and AHK1 like subfamily, all the subfamilies showed a monocot-dicot alternating pattern indicating the existence of these subfamilies before the divergence of monocots and dicots (Fig. 2). The phylogenetic analysis of the HPT members suggests 3 groups and 7 divisions. Phylogenetic analysis also suggested that all the *Musa* spp. HPTs can be clearly divided in three clades, I, II and III. It is interesting to note that 2 out of 3 clades are monocot specific (Fig. 2).

Analysis divided RRs into four groups namely Type-A RR, Type-B RR, Type-C RR and pseudo-RR. The phylogenetic tree of banana RRs with 7 other plants shows 3 groups and 6 divisions. All the RR group members clustered into their respective sub divisions and formed subgroups. Type-A RRs of *Musa* formed 3 clear subgroups, in which clade II and III were monocot-specific. Similarly, Type-B RRs of *Musa* formed 5 distinct groups as in other plants [7, 15, 19]. Interestingly, the Type-B RR members, MaRR34, MaRR35, MbRR21 and MbRR22, formed a distinct major clade with 1 Type-B RR of rice and dicot PRRs. Interestingly, no Type-B PRR member was identified in banana through phylogenetic analysis (Fig. 2a).

### Chromosome distribution and duplication in TCS members

The chromosomal localisation analysis of the TCS genes in both *M. acuminata* and *M. balbisiana* suggests that these are distributed throughout the genome. Though there is high level of synteny between the *Musa* spp., there were subtle differences in the localisation of the genes. A clear and distinct difference was localisation of higher number of TCS genes on *M. balbisiana* chromosome 12 as compared to same chromosome of *M. acuminata* (Additional file 2: Figures S2-S3). Structure analysis of CHK/HKs did not show much difference within the *Musa* spp. However, differences in the HPT gene structure could be clearly observed with comparatively higher number of introns present in the HPT genes in *M. balbisiana*. A clear difference between the number and size of exons were observed between the *M. acuminata* and *M. balbisiana* RR-Type-A, where comparatively the exons of *M. balbisiana* were much larger than that of *M. acuminata*. While comparing the Type-B-RR of *M. acuminata* and *M. balbisiana*, there were no such differences between the gene structures. While comparing the PRR groups, again a clear difference between the gene organizations was observed with comparatively larger introns in MbPRRs as compared to MaPRRs (Additional file 2: Figure S10).

To understand the effect of divergence on TCS genes, dN/dS analysis was performed. The analysis revealed peaks of Ks values with speciation time of A and B genomes (4.6 MyA, 0.04 Ks) and Ks of duplicated genes (0.5 Ks, 61 MyA). Result of dN/dS analysis showed almost an overlapping distribution pattern of Ks value for Ma-Ma and Mb-Mb (Fig. 2).

### *Cis*-regulatory elements in promoters of genes encoding TCS components

The analysis of the promoter region provides the information about the regulatory motifs present in genes. In our analysis, majority of CHK/HK genes showed the presence of various light-responsive as well as hormone-responsive motifs including GARE_motif (gibberelin), TCA element (salicylic acid), ABRE (ABA responsive), ERE (ethylene responsive) and TGA element (auxin responsive). Apart from these, the presence of heat (HSE), cold (LTR), drought (MBS, MYC) and pathogen responsive (TC-rich repeats) elements were also observed. ETR/ERS/EIN and PHY groups showed the presence of the hormone-responsive motifs with presence of elements related to wounding, biotic stress, elicitor and abiotic stress responses (Additional file 1: Table S2). In HPTs and RRs, various hormone-responsive and stress (biotic and abiotic) related motifs were observed. The presence of these elements was also observed in other plants [19, 21, 22]. Apart from these common *cis*-elements, a large number of transcription factor binding motifs were also observed in promoter sequences of genes encoding CHK/HK, HPT and RRs, which consist of C2H2, AP2/RAV, AP2/ERF, bHLH, Doff, MYB/SANT, MADS, Dehydrin, TCP, LEA, WRKY and alpha amylase (Additional file 1:Table S3). A number of novel motifs were also detected in the promoter region of TCS genes (Additional file 1: Table S4). Presence of these *cis*-regulatory elements in promoter regions provides a clue that the mode of action of TCS genes is not only depends on hormonal cross-talk but also triggers the other genes responsive to ripening or stress (biotic and abiotic) conditions.

### Expression of TCS genes during fruit development and ripening

The expression of the genes encoding members of TCS family members was studied using publicly available transcriptome databases of banana [11]. For ripening related studies, the transcriptome data available on the banana genome hub [11] and our in-house data [23] were used. The transcriptome datasets of 40, 60 and 90 DAF (days after flowering), and 40 DAF + acetylene, 60DAF + acetylene and 90DAF + acetylene for the fruit pulp were available on the banana genome hub. These datasets were used to analyse the effect of acetylene on various stages of fruit development. The in-house data on ripening was of unripe and ripe banana fruit pulp. Our analysis revealed that during fruit development, many of the TCS genes are not expressed. Only a few members of the HK class showed expression during fruit developmental stages. However, *MaCHK2.B*, *MaCHK2.A*, *MaCHK1.B*, *MaPHYA.A*, *MaERS1.A* and *MaETR1.C* were more than 2-fold up-regulated during ripening. In addition, analysis revealed down-regulation of *MaCHK2.D*. The up-regulation of *MaERS1.A* and *MaETR1.C* might be due to the increased activity of the ethylene signal transduction pathway during the ripening of the fruit. No significant change in expression of the HPT genes was observed during fruit development and ripening. The enhanced expression of *MaCHK2.B*, *MaCHK2.A* and *MaCHK1.B* could be due to the involvement of cytokinin during senescence, as ripening ultimately advances towards senescence (Additional file 2: Figures S4-S7).

**Fig. 3:**
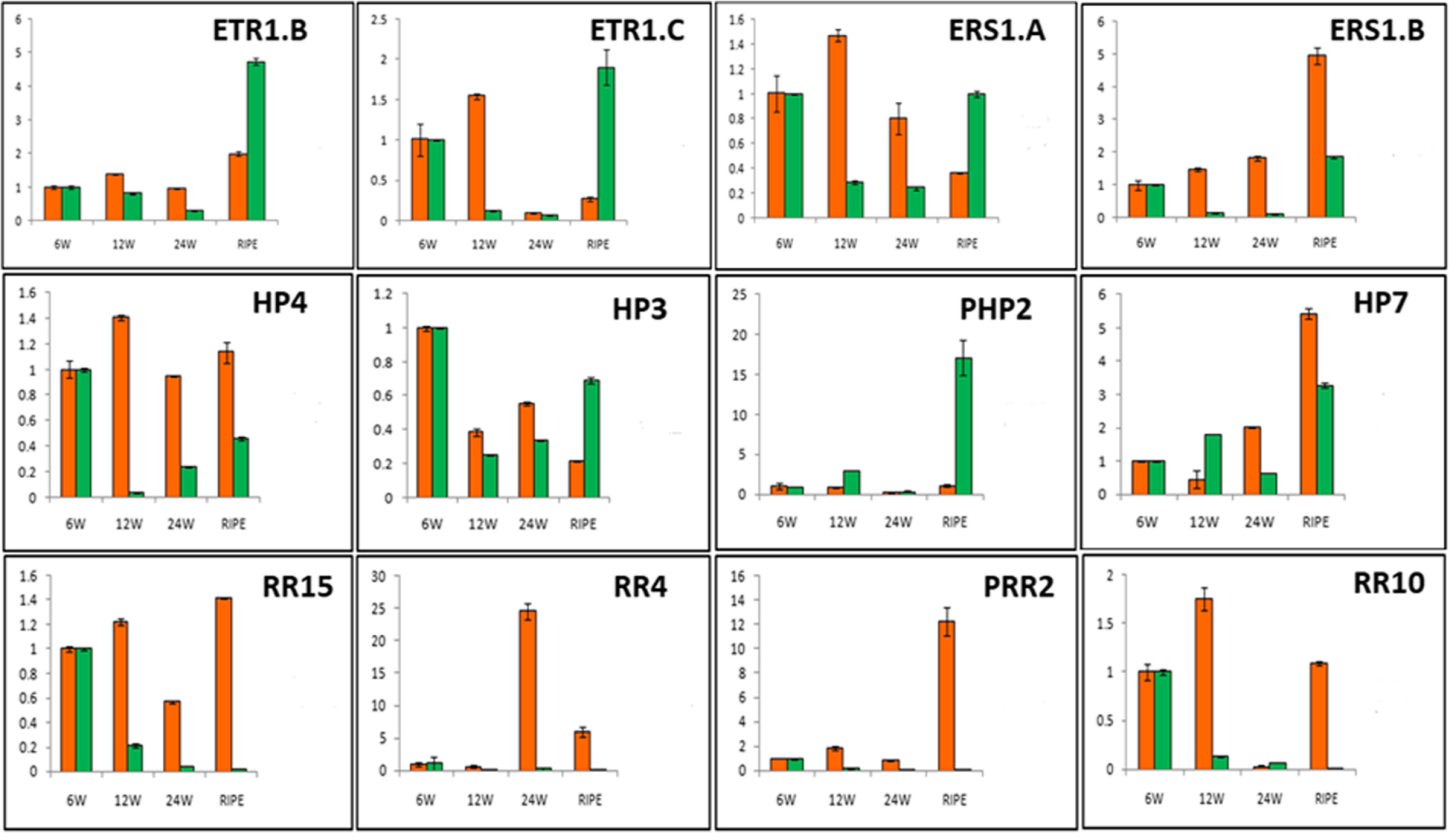
Expression of TCS genes during ripening. Expression profiles of TCS genes in fruit of dessert and cooking varieties. Orange and green bars represent dessert (*M. acuminata,* AAA genome) and cooking (*M. paradisiaca,* AAB genome) varieties respectively

Expression analysis revealed that a group of RR genes of Type-A and B express during the fruit development stages (Fig. 3). At least six Type-A RR (*MaRR2, MaRR4, MaRR6, MaRR8, MaRR14*, and *MaRR16*) genes were observed to express during fruit development stage as compared to four during post-harvest ripening (*MaRR4, MaRR7, MaRR8, MaRR15*). Four Type-B RRs (*MaRR24, MaRR26, MaRR27*) genes were found to be up-regulated during fruit development stage as compared to two (*MaRR25, MaRR32*) during post-harvest ripening. Analysis also revealed a significant difference in the expression of genes encoding PRRs during fruit development and post-harvest ripening (Additional file 2: Figures S4-S7). This pattern of expression of PRR genes, during ripening process was also observed in tomato and cucumber PRR genes [21, 22] which clearly indicate the role of PRR genes in ripening process.

Differential gene expression observed using transcriptome datasets was validated through Real-Time PCR analysis for selected genes using dessert (ripening) and cooking (non-ripening) banana varieties. Ethylene treatment of dessert variety let to enhance expression of *MaERS1.B* several fold as compared to cooking variety. Interestingly, *MaERT1.B, MaETR1.C and MaERS1.A* showed relatively higher expression during ripening in cooking variety compared to dessert variety. In HPT group, *MaHP4* and *MaHP7* genes exhibited the higher expression during ripening in dessert variety while *MaPHP2* and *MaHP3* showed relatively higher expression in the cooking variety. In RR group, *MaRR15* and *MaPRR2* showed high and *MaRR10* moderately higher expression during ripening in desert variety..

### Co-expression network during ripening

For better understanding of the mode of functional interaction of TCS genes, a co-expression network based analysis was performed, by calculating their Pearson’s correlation coefficient (r) values with the top 100 up- and down-regulated genes during fruit ripening. The co-expression network during ripening revealed the presence of genes encoding expansins, pectin-lyases, O-methyltransferases, enoyl coA hydrolase A8, Horcolin, Pathogenesis related protein, early-nodulin. These genes are known for their direct role in ripening as well as stress [24, 25].

**Fig. 4:**
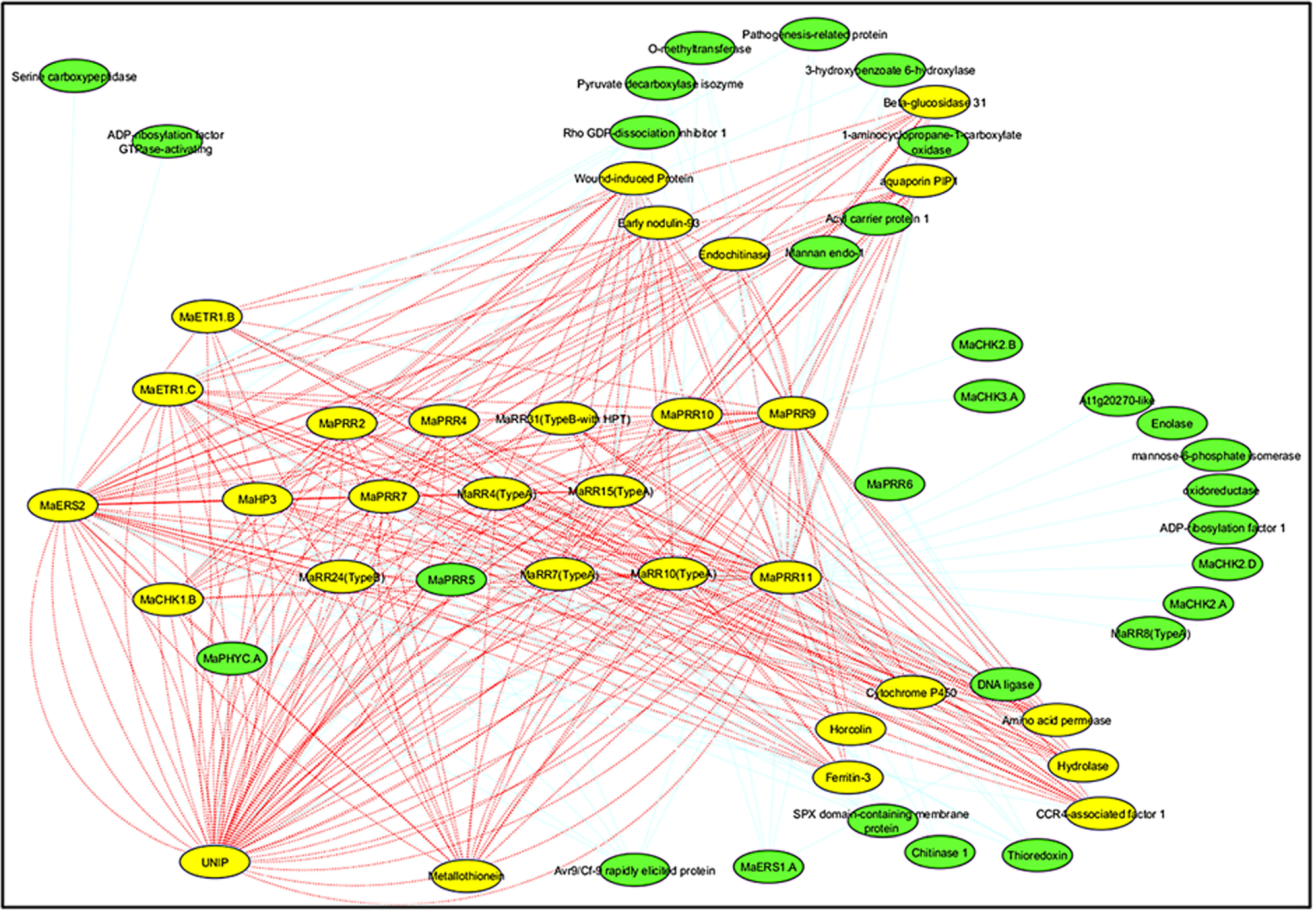
Coexpression network of *MaERS2* gene. Directive co-expression network of *MaERS2* gene, based on relative correlation coefficient (r value), during ripening process, where yellow nodes are direct contact and green nodes are indirect contacts

Since *MaERS2* was shown to be up-regulated during ripening in banana, genes that were co-expressed with *MaERS2* were analysed and a co-expression network was constructed. Around 29 direct contact modules could be identified through this analysis. Interestingly, a direct interaction module of *MaERS2, MaHP3, MaPRR, MaRR4* and *MaRR15* was clearly observed (Fig. 4). Direct interaction with other ethylene receptors also indicated the role of these receptors during fruit ripening in banana. To further explore the interaction of the ERS2 with HP and RR genes the molecular docking and dynamics analysis was carried out.

### Molecular docking and molecular dynamics analysis for TCS genes

As indicated by the co-expression results and recent reports [26], TCS genes regulate the signal transduction process, for different conditions, in a conserved pattern. To find the interactional specificity of TCS proteins with each other, molecular modelling based molecular docking and dynamics analysis method was used, which are known to analyse, such kind of interactions [27]. CHK, ERS, ETR and EIN proteins were used for the in silico analysis. In step one, the structure of receivers (CHK, ERS, ETR and EIN) were docked to the mediator (HPT) group protein. The docking results showed strong interaction between MbEIN4-MbHP2, MbERS1-MbHP2, MaETR1-MaHP1 (Table 1, Additional file 2: Figure S9).

**Table 1:** Interactions of different components of TCS complex in banana

Receptor	Primary Ligand	Energy (Kcal/mol)	Secondary Ligand	Energy (Kcal/mol)	Contacts	Binding Pattern
MaCHK1	MaHP1	− 816.12	MaRR1	−414	32	HPT
			MaRR21	− 143	20	HPT
			MaPRR1	−711	3900	CHK
MaERS1	MaHP1	− 532.39	MaRR1	− 815	134	HPT,HK
			MaRR21	− 882.51	370	HPT,HK
			MaPRR1	− 852.35	105	CHK
MbERS1	MbHP2	− 836.39	MbRR1	−531.23	224	HPT
			MbRR21	− 789.3	110	HPT,HK
			MbPRR1	− 415.29	11,579	HPT,HK
MaETR1	MaHP1	− 829.27	MaRR1	− 772.59	86	HPT,ETR
			MaRR21	− 925.56	202	ETR
			MaPRR1	− 637.2	32	ETR
MbETR1	MbHP2	−816.5	MbRR1	− 576.6	102	HPT
			MbRR21	− 3280.0	8899	HPT,ETR
			MbPRR1	− 538.9	189	HPT,ETR
MaEIN4	MaHP1	− 763.03	MaRR1	− 853.65	71	HPT,EIN
			MaRR21	− 719.01	68	EIN
			MaPRR1	− 629.6	1562	EIN
MbEIN4	MbHP2	− 924.03	MbRR1	−688.24	123	HPT
			MbRR21	− 850.1	187	EIN
			MbPRR1	− 439.8	60	HPT

In step two docking, the primary docking complex was docked with the RRs (Type-A, Type-B and PRR) for which one representative protein from each group were taken for the interaction. Results of the secondary docking revealed a pattern of interaction between primary complex and MaRR1, MaRR21 and MaPRR1. MaCHK1-MaHP1 complex interaction with RR, exhibited the best interaction with PRR1 protein having ∆G of − 711.6 with 3900 contacts, on the other hand interactions with RR1 and RR21 showed comparatively higher ∆G (− 414 and − 413 respectively) with lesser number of contacts (32 and 20 respectively). MaERS1 and MbERS1 showed an interesting interaction pattern with RRs, where both proteins from *M. auminata* and *M. balbisiana* showed the best interaction with Type-B RR (MaRR21) in terms of ∆G. A similar pattern of the interaction was observed between the MaETR1 and MbETR1 where both the proteins again interacted with the Type-B RR (MaRR21). Interaction of MbEIN4 protein also preferred the MbRR21, which is a Type-B response regulator, but the MaEIN4 selectively preferred the Type-A regulator MaRR1 in terms of energy. Interestingly, MaPRR1, MaEIN4 formed the complex with large number of contacts (1562) (Table 1, Additional file 2: Figure S9).

To check this different interactional pattern, an all atom molecular dynamics simulation was performed for the CHK-HPT complex, CHK-RR and CHK-HPT-PRR complex in water with and without ethylene for the time range of 50 ns. For the accuracy of results, previously modelled structures were mapped on the reported PDB structure files of TCS component proteins of *Arabidopsis* (PDB ID 4EUK, 3T4J, 4PAC). *Musa* sequences were edited and then again modelled and minimized by MD to reduce the structural errors of in silico protein model and structural gap fillings, using templates of Arabidopsis TCS pdb’s (PDB ID 4EUK, 3T4J, 4PAC).

**Fig. 5:**
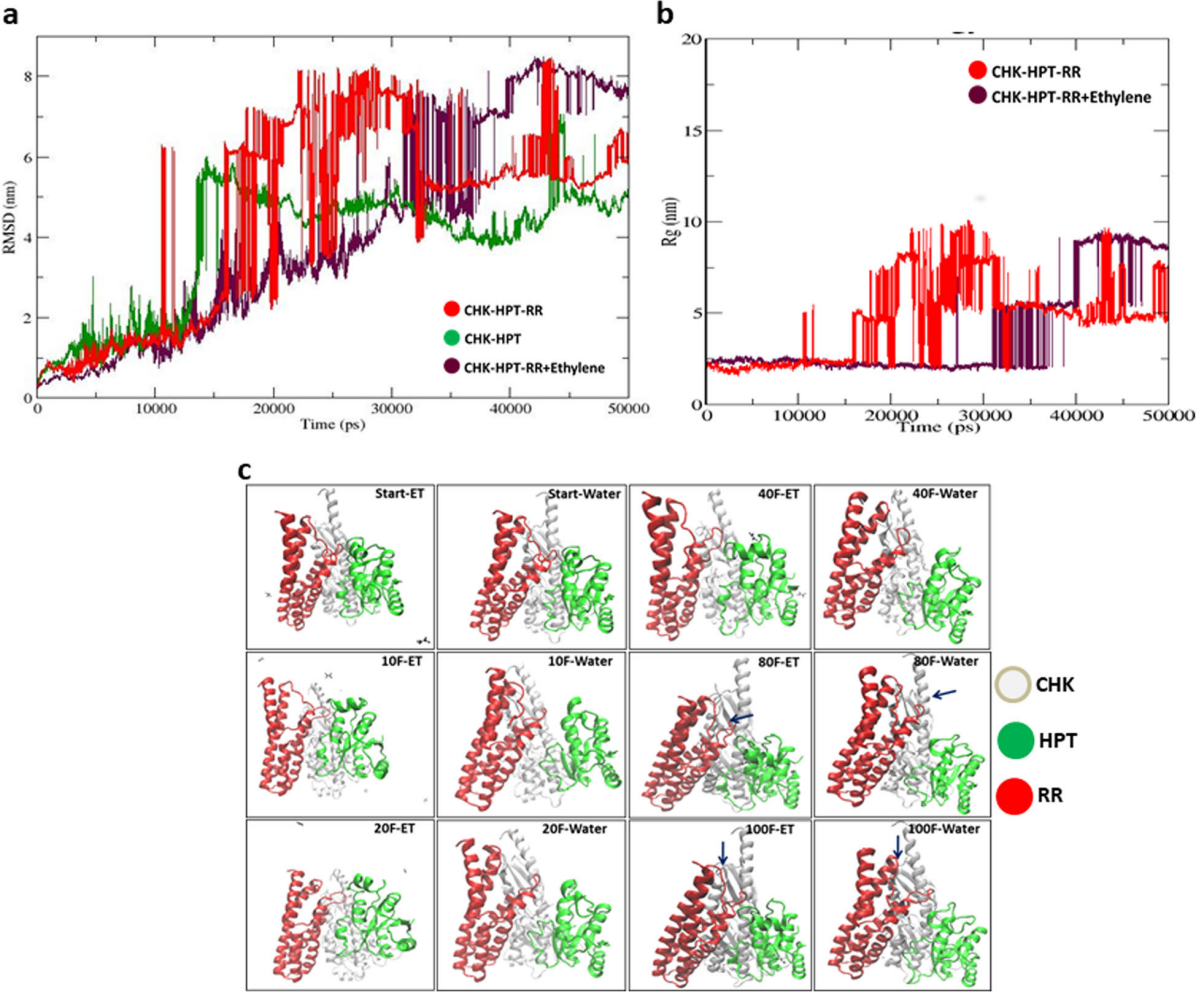
Comparative molecular dynamics analysis of TCS protein complex with or without ethylene. **a** Represents comparison between CHK-HPT **+** CHK-HPT-RR complex and CHK-HPT-RR + Ethylene complex in their scalar distance between atoms. **b** Presents comparison between CHK-HPT + CHK-HPT-RR complex and CHK-HPT-RR + Ethylene complex in their structural compactness by their gyration radius. **c** Structural changes in TCS complex with and without ethylene. Frame wise comparison between CHK-HPT-RR complex and CHK-HPT-RR + Ethylene complex, obtained after 50 ns production MD run, to show the structural changes in protein complex during presence of ethylene and without ethylene. CHK represented by white/silver coloured structure, HPT by green and RR by red. Minute background structures are ethylene

The modelled structures were divided into 3 parts, first CHK + HPT complex, representing the initial complex, second CHK + HPT + PRR (water) representing the basic two component machinery and third CHK + HPT + PRR (ethylene) to represent the condition of TCS complex in presence of ethylene. The first complex, CHK + HPT, showed compact conformation, although many fluctuating peaks were observed, it maintained its structural compactness. We assume that both, CHK and HPT, may be recognising their structural folds, with a decent number of H-bonds for further function. It also indicates that during fold recognition, TCS receptors decide the suitable mediator according to the condition for further process. Our second system, CHK + HPT + PRR(W) shows a highly dynamic complex, with many fluctuating peaks, which clearly indicates that primary complex is facing highly strong distance based pulling. It can be hypothesized that primary complex may be trying to recognise the suitable response regulator to complete the main TCS machinery for a specific functions. Such recognition patterns are already reported in *Arabidopsis* for cytokinin dependent signalling [28]. During the main complex formation, constant fluctuations in rms distance also indicates towards the same phenomenon with an indication that probably at this step of interaction HPT decides or guides for the selection of suitable PRR (Fig. 5).

After these two systems, it was important to study the dynamic behaviour of the TCS complexes with ethylene in the system. For this, 6 molecules of ethylene were inserted in 50,000 molecules of water for a concentration of 12 ppm, to match the concentration of ethylene during ripening. Analysis revealed a striking difference between the dynamics of the complexes. Insertion of ethylene clearly stabilized the complete complex, which is reflected in rms distance and gyration plots. The visualization of the TCS complex at different stages suggested changes in conformation of HPT and RR in the presence of ethylene. The analysis also revealed that HPT is facilitating RR in getting optimum conformational geometry. This shows that ethylene plays a pivotal role in maintaining not only the structural stability but also the conformational selectivity, as one ethylene molecule was in close proximities of HPT throughout during its interaction with RR helices. The analysis also suggests that concentration of ethylene plays a critical role in conformational pattern recognition, which further activates the selectivity of primary complex towards certain response regulators.

Another comparison for structural preference was made between CHK + HPT and CHK + PRR complexes. Although the scalar distance of CHK + PRR complex was better than CHK + HPT, both complexes showed a similar pattern of trajectory in rmsd plot, after 12 ns. On the other hand, comparison of structural compactness shows better steady state for CHK + PRR complex than the CHK + HPT complex. This result supports the hypothesis that HPT is playing a role in guiding or preferential binding for response regulators. Number of H-bond is also higher in CHK + HPT complex instead of CHK + PRR complex.

## Discussion

The TCS is one of the most evolutionarily conserved signalling modules, encoded by multigene families and present in all living organisms. Studies suggest that this signalling cascade plays an important role in various plant processes including plant development and various stress responses [8]. The members of this family have been identified in several plants however; no effort has been made to identify members of this signalling module from banana. In this study, we identified 80 TCS (25 HK, 10 HPT and 45 RR) gene family members in *M.acuminata* and 72 (25 HK, 5 HPT and 42 RR) in *M. balbisiana*. The TCS members of *M. acuminata* and *M. balbisiana* shared higher sequence identity with rice and maize as compared with Arabidopsis suggesting monocot-specific structural and functional conservation of this family of proteins. Musa spp. encodes the largest number of TCS gene as compared to other monocots. This increase in number might be due to whole genome duplication events during evolution [11]. As members of this gene family have specialised roles in responding to various environmental and developmental cues and pass on the signal to the response regulators, increase in the number of the HK family enhances the plant’s specificity for various responses. Our analysis suggested that TCS genes are distributed throughout the genomes of *M. acuminata* and *M. balbisiana* and show high level of synteny. However, a clear and distinct difference was observed in the localisation of higher number of TCS genes on *M. balbisiana* chromosome 12 as compared to same chromosome of *M. acuminata*.

The domain analysis of the HK proteins showed the presence of the conserved domains with a few exceptions. The CHK proteins have extra TM domains in *M. balbisiana*. The CHASE domain is an important region for the recognition and binding of cytokinin and proteins having this domain have been shown to be involved in cytokinin perception and signalling. Although CHK proteins of *M. acuminata* and *M. balbisiana* had similar domain architecture as reported for other plants, these showed the presence of higher number of CHK proteins (9 in both, *M. acuminata* and *M. balbisiana*) in comparison with other reported plant CHKs (Additional file 1:Table S1). As cytokinins play a major role in the shoot multiplication, the higher number of CHK genes in *Musa* spp. may have an important role in shoot multiplication for the propagation. The CHKs of *M. acuminata* and *M. balbisiana* share 30–40% sequence identity with *Arabidopsis* whereas higher sequence identity with rice (60–80%) and maize (50–70%). This reflects monocot-specific structural and functional conservation in this family of proteins. In addition, the diverged domain organization was also observed in MbERS1.D and MbERS2 in relation to TM and GAF domains. Analysis also suggested that MaERS1.A lacks the domain essential for ethylene binding and subsequent signalling cascade and may not be playing an important role in the ethylene perception. The MbHP7 had TM, P450 and STAT_int domains which play important role in innate and host acquired immune responses. The PHY proteins in *M. acuminata* and *M. balbisiana* did not show divergence from the standard PHY proteins indicating functional conservation.

In our study, we identified a total of 46 and 42 RR genes in *M. acuminata* and *M. balbisiana* respectively and classified in 4 different groups*.* Though this family showed conservation in domain structure, a few differences were observed in *M. acuminata* and *M. balbisiana*. In *M. balbisiana*, the Type-B sequences contained additional functional domains which are mostly related to the apoptosis or defence against stress/pathogen. Presence of these domains indicates the responsiveness/sensitivity towards defence response during different stress conditions. It also indicates their possible role in initiating/regulating ethylene mediated defence pathway in *Musa* plants.

The cis-regulatory analysis showed the presence of various light- responsive as well as hormone-responsive motifs in the promoters of the TCS genes. Most importantly, ETR/ERS/EIN and PHY groups contained the hormone-responsive motifs with the presence of elements related to wounding and stress responses. The presence of these elements has already been shown in the promoters of genes related to TCS family in other plants [21, 22]. Apart from these common *cis*-elements, a large number of known and novel motifs were identified in different TCS genes (Additional file 1:Table S3, Table S4). Presence of these *cis*-regulatory elements in promoter regions provides a clue that the mode of action of TCS genes is not only depends on hormonal cross-talk but also triggers the other genes responsive to ripening or stress (biotic and abiotic) conditions.

The expression of the genes encoding members of TCS family members during fruit development and ripening was studied using publicly available transcriptome databases of banana [11] and our in-house data [23]. Our analysis revealed a clear difference in the expression of TCS genes during fruit development and ripening. This differential pattern of expression of TCS genes, during fruit development and ripening process was also observed in tomato and cucumber PRR genes [21, 22]. Our analysis also indicates that probably TCS genes have some pre-defined co-expression network for biotic and abiotic stress conditions as well as during ripening.

These genes have specific signal receiver (CHK/HK, ETR/ERS/EIN, PHY) for different conditions as well as specific mediators (HPT) and regulators (RR). These results also indicate that probably TCS genes have some pre-defined co-expression network for biotic and abiotic stress conditions as well as during ripening. Ripening marks several changes in the physiological and biochemical attributes of fruit. Several hundred genes are recruited to express differentially during this process and their cumulative effect brings about ripening and softening in the fruit. Previously, using suppression subtractive hybridization (SSH), we identified 37 EST-unigenes from banana which are expressed differentially during ripening. About 50% of these belong to processes such as stress, defence and detoxification. Besides these, we have also identified genes which are known to be involved in the regulation of gene expression and other processes, although their expression has not been reported during fruit ripening. Expression studies clearly suggest that the most of these genes are ethylene-regulated and related to banana fruit ripening. It is concluded that ethylene-induced banana ripening evokes a stress-like response and several genes belonging to stress/defence are expressed in addition to genes related to ethylene biosynthesis, cell wall hydrolysis, secondary plant product biosynthesis, fatty acid biosynthesis, metabolite transport and transcription/translation machinery. The ripening network showed that a number of genes which influence different steps of fruit maturation are in direct proximity with two component proteins. Moreover, stress-responsive genes, present in same network, clearly indicate towards the bi-functional signalling activity of TCS proteins, which may be guided by the ethylene stimulus (Additional file 2: Figure S8). Gene co-expression networks suggested a direct role of the TCS module during ripening and a number of genes related to cell wall modification, aroma, and ethylene signal transduction were found to be differentially regulated and associated with the network. These genes have previously been shown to be up-regulated during ripening and fruit development [11, 23].

To find the interactional specificity of TCS proteins among members, molecular modelling based molecular docking analysis was carried out. Analysis suggested a strong interaction between MbEIN4-MbHP2, MbERS1-MbHP2, MaETR1-MaHP1. MaERS1 and MbERS1 showed an interesting interaction pattern with RRs, where both proteins from *M. auminata* and *M. balbisiana* showed the best interaction with Type-B RR (MaRR21). A similar pattern of the interaction was observed between the MaETR1 and MbETR1 where both the proteins again interacted with the Type-B RR (MaRR21). The docking analysis showed that in cases where PRR proteins interact with the receiver-mediator complex, the PRR domains bind with the receiver/catalytic domain. However in cases of RR interactions, bindings were observed with the receiver-mediator complex or HPT. Such interactional pattern provides clues about the specificity of PRR toward the receivers and indicates that PRRs have a specific binding for the complex.

Our analysis also suggested specificity of PRR toward the receivers and indicates that PRRs have a specific binding for the complex. We also hypothesize that primary complex may be trying to recognise the suitable response regulator to complete the main TCS machinery for a specific functions. Such recognition patterns are already reported in *Arabidopsis* for cytokinin dependent signalling [28]. In addition, analysis revealed a striking difference between the dynamics of the complexes. Insertion of ethylene clearly stabilized the complete complex, which is reflected in rms distance and gyration plots. We presume that this mechanism is followed in other stress conditions where other molecule plays a similar role. Similar analysis has been performed in the InsP interaction with the Jasmonate receptor [29]. In this study, it was observed that the binding of InsP with COI1 stabilises the conformation of COI1 and promotes the binding between JAZ1 and COI1. Several specific amino acid residues have also been identified in these molecules which play an important role in the stability of this receptor complex. The molecular docking and dynamics study has also been applied to the study the mechanism of auxin interaction with auxin-binding protein [30]. The observed pattern indicates the nature of TCS signalling, which requires suitable mediators which are preferred for signal mediation to final step executors. These interaction patterns also show that the specificity towards any protein binding site is condition driven and mediators or response regulators may differ in different developmental or stress conditions.

## Conclusion

In this study, the members of TCS gene family were identified from *M. acuminata* and *M. balbisiana*. Though members of these families are highly conserved, subtle changes in the structure of the receptors and regulators may play an important role in the divergence of this TCS module in *Musa* spp. The phylogenetic analysis suggests that the whole-genome duplication has played an important role in the expansion of this gene family in *Musa* spp. The co-expression network showed a direct interaction between receptor, histidine kinase and the response regulator. This was further confirmed by the molecular docking and dynamics analysis. Though validation of these interactions is required, analysis suggests specific interactions of TCS members during fruit ripening process which are regulated by ethylene.

## Methods

### Identification of TCS members in banana

The TCS family members of banana were identified by using the *Arabidopsis* and rice sequences as input seeds in *M. acuminata* and *M. balbisiana*, applying a 3 step identification process. In step 1, BLAST program [31] with e-value 5 was used to find out the TCS genes in *Musa* sequence database, in step 2 a profile Hidden Markov Model [13] was constructed and sequences were searched on the basis of profile HMM followed by search of TCS genes by function in Banana Genome Hub (http://banana-genome-hub.southgreen.fr/) [32] in third step. All the retrieved sequences were checked by the SMART database (http://smart.embl-heidelberg.de/) [33] and CDD [34] for the presence of conserved TCS domains. Sequences lacking the conserved TSC domain were excluded from further analysis.

### Gene structure, multiple sequence alignment, phylogenetic analysis & conserved motif search

Gene structures of the TCS genes were demonstrated using the Gene Structure Display Server (GSDS) [35]. Identified sequences of the TCS proteins from *M. acuminata* and *M. balbisiana* were retrieved from Banana Genome Hub Database, conserved TCS domains CHK, HK, HPT, Rec, MYB and CCT were then aligned using Clustal X [36] with gap opening penalty of 10 and gap extension penalty of 0.1. Phylogenetic relations of banana TCS with rice and *Arabidopsis* were analysed using MEGA7 [20] tool, maximum likelihood method with the JTT model for amino acids having bootstrapping value 1000, were considered as the parameters. To find out the conserved de novo sequential motif in TCS sequences, offline MEME [37] program was employed.

### RNA isolation and expression analysis

Dessert (*M. acuminata*) and cooking (*M. paradisiaca*) varieties of banana were used for the gene expression analysis. *M. paradisiaca* is the hybrid of *M. acuminata* and *M. balbisiana* belonging to the AAB genome. Fruits from three independent plants at different developmental stages from each cultivar were harvested to collect the samples. Banana fingers from the same whorl of the hand representing similar developmental stage were treated with 100 μL/L ethylene for 24 h at 22 °C in dark and then allowed to ripen in air as described in Lohani et al. [38]. Other vegetative tissues were harvested from six-month old banana plants growing in the field. The sampling was repeated two times at different time intervals to cater for seasonal variation. For RNA isolation, plant materials were quickly frozen in liquid nitrogen before extraction or stored at − 80 deep freezer for further use.

Total RNA were isolated from banana tissues according to previously described protocol [39]. Each RNA sample was treated with DNase I Digest kit (Sigma-Aldrich, USA) to eliminate DNA contamination. The integrity and size distribution of total RNA was analysed by agarose gel electrophoresis. A NanoQuant (Infinite® 200 PRO NanoQuant, Austria) was used for RNA quantification. DNA-free RNA (5 μg) was used for synthesis of first strand of cDNA by using Revert Aid First Strand cDNA synthesis Kit (Fermentas, USA) as per manufacturer’s recommendations. The quantitative Real-Time PCR expression was carried out with an ABI 7700 Sequence Detector (Applied Biosystems, USA). The transcripts were quantified by SYBR Green chemistry. The amount of cDNA was normalized by using amplification of housekeeping banana actin as an internal control. A list of primers used for expression analysis of specific genes is provided in Additional file 1: Table S5. The data from Real-Time PCR amplification was estimated in terms of comparative fold expression following 2^−∆∆ct^ method. Expression analysis was carried out using three biological replicates. Each reaction was performed in 20 μl (total volume) and consisted of 1X SYBR Green Master mix (Applied Biosystems, USA), 5 pmol of each primer, 1 μl cDNA template and sterile H_2_O. The steps performed during real-time PCR experiment were as follows: step (1) 50 °C, 2 min; step (2) 95 °C, 10 min; step (3) (95 °C, 0.15 min; 60 °C, 1 min) × 40 cycles.

### Promoter region analysis of TCS genes in *Musa* spp.

The 2000 bp upstream regions from the translation start codon (ATG) of *Musa* TCS genes were selected for the putative promoter region analysis. A two-step process was carried out to search for the presence of phytohormone and biotic as well as abiotic stress related *cis*-regulatory elements. In step 1, the promoter sequences were analysed in PlantCARE [40] and PLANT PAN [41] online tools for the presences of motifs. In step 2, known phyto-hormone responsive and stress related elements were searched in retrieved putative promoter sequences.

### Chromosomal localization, gene duplication and evolutionary analysis in *Musa* spp.

The chromosomal position of retrieved TCS genes were physically mapped on 12 *Musa* chromosomes using the gene position coordinates obtained from Banana Genome Hub. Segmental duplicates were identified using the synteny analysis, which has been performed using MCScanX tool [42]. The selective pressure on duplicated gene pairs and the occurrence of duplication events with their time of divergence were estimated by calculating the synonymous substitution (dS) and nonsynonymous substitution rate (dN), using the PAL2NAL [43] online tool (http://www.bork.embl.de/pal2nal/).

### Protein structure modelling, docking simulation, gene co-expression network analysis

For better insight of TCS proteins interaction and co-expression patterns, in silico structures were modelled using Phyre2 [44] online tool. Phyre2 tool was used with many bacterial as well as plant protein structures as template. To increase the accuracy of modelled structures, proteins were mapped on the plant TCS structures (PDB ID 4EUK, 3T4J, 4PAC) as homologs due to higher structural coverage. These structures were further minimized for 2 ns in energy minimization step in Gromacs-4.5.6 [45] tool using steepest descent method, minimized structures were further used in docking simulation. Protein-Protein docking of TCS proteins, performed by using HEX [46] tool. After docking, the complex of 3 proteins was used to create the MD system with and without ethylene. The coordinates of structure and trajectory of ethylene were generated using ATB [47]. These 2 systems were again minimized and underwent the all atom MD simulation of 50 ns. As these proteins are very specific in their function during different treatments, there co-expression networks were generated using CoExpNetViz [48] module with r-value 0.5 of Cytoscape [49] tool which were further viewed and analysed for their interaction pattern in Cytoscape [49] .

## Additional files

Additional file 1: **Table S1.** Description of members of TCS gene family in different plants. **Table S2.** Motifs identified in promoter sequences of TCS genes in banana using PlantCare online tool. **Table S3.** Transcription factors identified in promoter region sequence of TCS genes of banana using Plantpan online tool. **Table S4.** De novo motifs identified in promoter region sequence of TCS genes using MEME tool. **Table S5.** Primer sequences of selected TCS genes for the qRT PCR analysis. (RAR 343 kb)

Additional file 2: **Figure S1.** Domain architecture of TCS proteins in *Musa acuminata* and *Musa balbisiana* obtained using SMART domain detection tool. **Figure S2.** (A) Circular representation of gene duplication event of TCS gene family in *Musa acuminata* and *Musa balbisiana*, generated using reciprocal blast hits for tandem and segmental duplicates in MCScanX program. (B) Circular representation of gene duplication event with in TCS gene groups in *Musa acuminata* and *Musa balbisiana*, generated using reciprocal blast hits for tandem and segmental duplicates in MCScanX program. **Figure S3.** Bar representation of chromosomal position of TCS genes in *Musa acuminata* (A) and *Musa balbisiana* (B). **Figure S5.** Expression of RR-A, RR-B and PRR genes in ethylene treatment: (A), (C) and € represents Log2 transformed fold change values from Banana Genome Hub DB, for 40 days, 60 days, 90 days (normal and ethylene treatment). (B), (D) and (F) represent Log2fold change value for TCS genes from 4 day ripe pulp (after ethylene treatment). **Figurfe S6.** TCS gene expression analysis in fungal infection in corm and roots of banana plant, expression of Receptor CHK/HK and mediator HPT genes in fungal infection: joint panel of 3 groups represent the fungal infection in root at 3 h, 27 h, and 51 h by Foc1 & Foc4 strains. Corm represents Log2 transformed fold change values for receptors during infection in corm. **Figure S7.** Expression of RR-A, RR-B and PRR genes in fungal infection: Joint panel of 3 groups represent the fungal infection in root at 3 h, 27 h, and 51 h by Foc1 & Foc4 strains. Corm represents Log2 transformed fold change values for receptors during infection in corm. **Figure S8.** Co expression network of TCS genes in ethylene treatment (A) and in fungal infection (B), based on correlation value (r) of TCS genes as bait, with top 200 differentially expressing genes. Cytoscape program was used for calculation and visualization of the network. **Figure S9.** Pictorial representation of docked proteins in TCS complexes, obtained by molecular docking. (A) Represents, representative members of receptor proteins with MaHP1 and MaRR1. (B) Represents, representative members of receptor proteins with MaHP1 and MaRR21. (C) Represents representative members of receptor proteins with MaHP1 and MaPRR1. **Figure S10.** Gene structure for HK, HPT and RR genes of *Musa acuminata* and *Musa balbisiana*, generated using GSDS online tool from using the genomic coordinates of banana GFF. (RAR 4103 kb)

## Data Availability

Not Applicable.

## Abbreviations

ABRE: ABA responsive element
CCT domain: Co, Col and Toc domain
CHASE: Cyclase/HK-Associated Sensory Extracellular
DAF: Days after flowering
ERE: Ethylene responsive element
EST: Expressed Sequence Tags
ETR: Ethylene Receptor
HK: Histidine Kinase
HMM: Hidden Markov Model
HPT: Histidine Phosphotransferase
MyA: Million years ago
PRR: pseudo-RR
RR: Response Regulators
TCS: Two-Component System
TM: Trans-Membrane
WGD: Whole Genome Duplication
